# Choice architecture modifies fruit and vegetable purchasing in a university campus grocery store: time series modelling of a natural experiment

**DOI:** 10.1186/s12889-018-6063-8

**Published:** 2018-10-01

**Authors:** Rosemary Walmsley, David Jenkinson, Ian Saunders, Tony Howard, Oyinlola Oyebode

**Affiliations:** 1 Warwick Medical School, University of Warwick, Gibbet Hill Campus, Coventry, CV4 7AL UK; 2 Worcester College, University of Oxford, Oxford, OX1 2HB UK; 3 Campus and Commercial Services Group, University of Warwick, Coventry, CV4 7AL UK; 4 Department of Computer Science, University of Warwick, Coventry, CV4 7AL UK

**Keywords:** Choice architecture, Nudge, Health behaviour, Behaviour change, Tertiary education, Young adults, Diet, Fruit and vegetables

## Abstract

**Background:**

In developed countries, adolescent and young adult diets have been found to be nutritionally poor. The aim of this study was to examine whether a choice architecture intervention, re-arrangement of produce within a grocery store to increase the accessibility of fruit and vegetables, affected purchasing behaviour on a university campus.

**Methods:**

A database of daily sales data from January 2012 to July 2017 was obtained from a campus grocery store. Two changes to the layout were made during this time period. In January 2015, fruit and vegetables were moved from the back of the store, furthest from the entrance, to the aisle closest to the entrance and an entrance-facing display increasing their accessibility. In April 2016, the entrance-facing display of fruit and vegetables was replaced with a chiller cabinet so that fruit and vegetables remained more accessible than during the baseline period, but less accessible than in the period immediately previously. A retrospective interrupted time series analysis using dynamic regression was used to model the data and to examine the effect of the store re-arrangements on purchasing. All analyses were carried out both for sales-by-quantity and for sales-by-money.

**Results:**

The first shop re-arrangement which made fruit and vegetables more prominent, increased the percentage of total sales that were fruit and vegetables, when analysed by either items purchased or money spent. The second rearrangement also had a positive effect on the percentage of total sales that were fruit and vegetables compared to baseline, however this was not significant at the 5% level. Over the five year period, the percentage of sales that were fruit and vegetables declined both in terms of items purchased, and money spent.

**Conclusions:**

Increasing accessibility of fruit and vegetables in a grocery store is a feasible way to improve the diet of students in tertiary education. There is evidence of declining fruit and vegetable consumption among the studied population, which should be further investigated.

**Electronic supplementary material:**

The online version of this article (10.1186/s12889-018-6063-8) contains supplementary material, which is available to authorized users.

## Background

Inclusion of fruit and vegetables in the diet is recommended for good health by the World Health Organisation [1] and by policy makers in many countries [2–4]. Meta-analysis of sixteen prospective cohort studies has demonstrated that increasing fruit and vegetable consumption is associated with lower mortality from all causes, cardiovascular disease, and cancer [5]. This has also been demonstrated for the UK population using nationally representative data from the Health Survey for England [6].

In developed countries, the diets of adolescents and young adults have been found to be nutritionally poor, and particularly so, in comparison with other age groups [7–9]. This is generally true across 28 countries in the European Union [7]. The Health Survey for England found that in 2015 16–24 year olds ate a mean of 2.9 portions of fruit and vegetables per day, compared to the mean for all adults of 3.5 portions per day [8]. Similarly, the 2015 Scottish Health Survey showed 2.6 portions a day were consumed by 16–24 year olds and 3.1 portions per day for all adults [9]. Taking into account the low current level of consumption in this age-group, and the potential health benefits, increasing fruit and vegetable consumption amongst young adults is a desirable public health goal.

In many developed countries, a large proportion of the population participates in tertiary education [10]. The UK government reports the Higher Education Participation Rate (the likelihood of a young person participating in Higher Education by age 30) as 48% in 2014/2015 [10]. In 2015/16, the Higher Education Statistics Agency reports 2,280,830 students in UK Higher Education (and a further 410,130 staff) [11–13]. Qualitative studies have found that students in tertiary education experience the increased independence noted as characteristic of young adulthood, and report greater autonomy over food choice than before beginning tertiary education [14–16]. Furthermore, participants in these studies report particular challenges of university environments including lack of time or facility for cooking and high availability of unhealthy choices in campus food outlets [14–16]. This is supported by evidence of unhealthier eating of students who live on campus compared with students living off campus in surveys in England [17]. There is also evidence that students living outside their family home have unhealthier diets than those living at home: this is reported among Greek students [18] and in a multi-country study of Bulgarian, Danish, German and Polish students [19]. It is unsurprising therefore to find multiple studies, principally in the US, but also in England, reporting a mean weight gain in students who have recently begun university study [20–23]. Those that compare students beginning university with their peers not beginning university found students beginning at university gained more weight, though studies vary in the size of effect reported [20, 24].

The UK government’s plan for action on childhood obesity notes that:

*“Every public sector setting, from leisure centres to hospitals, should have a food environment designed so the easy choices are also the healthy ones.”* [25]

The plan describes how these spaces should ‘set an example’ to children and families [25]. It is not clear how universities fit in to this strategy, as they receive significant amounts of public funding alongside private funding. However, it seems reasonable in this policy environment, that there will be expectation in the coming years that universities adopt some degree of health promoting activity. Looking beyond government policy, 88 UK Universities have signed up to ‘Healthy Universities’, a voluntary scheme under which they commit to the principles of the Okanagan Charter (an international charter for health promotion at universities and colleges), which includes the following call to action for higher education institutions:

*“Embed health into all aspects of campus culture, across the administration, operations and academic mandates.”* [26].

In light of participation rates and the association of university study with increasing autonomy, universities offer a setting in which public health interventions to reduce obesity and promote healthy diets may have an important effect.

‘Choice architecture’, sometimes termed ‘nudge’, is increasingly being considered as an effective tool to promote healthy behaviours [27]. Choice architecture in micro-environments (e.g. homes, workplaces) has been defined as:

“*Interventions that involve altering the properties or placement of objects or stimuli within micro-environments with the intention of changing health-related behaviour. Such interventions are implemented within the same micro-environment as that in which the target behaviour is performed, typically require minimal conscious engagement, can in principle influence the behaviour of many people simultaneously, and are not targeted or tailored to specific individuals”* [28]*.*

Interventions that altered the environment in tertiary education food outlets were found to improve diet according to various outcomes in a systematic review of the literature in 2015 [29] and interventions making healthy options more easily accessible have been found to increase healthy behaviour in a number of studies [30]. *Choice architecture* might be considered particularly apt for targeting young adults as it does not act by limiting choice, it respects the autonomy of young adults and their rights and capacity to make their own decisions in respect of health related behaviours. This is in contrast with what might be appropriate where the target population is children. There is also some evidence derived from qualitative research, that university students fail to engage with healthy behaviours because of perceptions of health problems as distant or their health as invincible [15, 31]. Thus choice architecture interventions, which may require minimal conscious engagement, may be more suited to this group than interventions requiring greater conscious engagement.

This study examined whether a choice architecture intervention, re-arrangement of produce within a grocery store, affected fruit and vegetable purchasing behaviour on a university campus.

## Methods

### Setting

The University of Warwick is a campus university on the edge of the city of Coventry, in the West Midlands of England, UK. As of 2017, it had around 26,000 students (of which around 15,000 were undergraduates) [32]. The Rootes grocery store (a branch of Costcutter) is the only on-campus grocery store and stocks a wide range of food. Over the study period, it had mean term time weekly takings of £142,177. There are other outlets on campus which sell ready-to-eat meals and snacks.

A change to store layout (Intervention A) was made for January 2015, providing an in-the-field opportunity to investigate the effect of proximity and ease of access on what was purchased in store. In particular this made fruit and vegetables more prominent in the store. Fruit and vegetables were moved from the back of the store, furthest from the entrance, to the aisle closest to the entrance and also an entrance-facing display. A further change (Intervention B) was made in April 2016, replacing the entrance-facing display of fruit and vegetables with a chiller cabinet containing drinks (juices, smoothies and sugar sweetened beverages). The first change (Intervention A) was made at the same time as a store renovation including changes to the store decoration and branding. The second change (Intervention B) was the only one made at this timepoint (Additional file 3).

### Data

A database of daily sales data from January 2012 to July 2017 was obtained from Rootes grocery store. The data contained the daily total quantity sold for each product, along with the product description, price, profit, barcode, unit size and the food category. These files were imported to R software (protecting the original files). Fields in the database of interest were selected: Sale Date, Category, Product Description, Price, and Quantity sold. Other variables were added to the data frame, indicating whether the day was a day of term time or outside of term time, the number of days from the beginning of the time period being studied, and which intervention period the date fell in (as two binary variables).

Code was written into which a category vector could be entered and R would return a data frame with quantity of sales made by a particular category aggregated by day. Although the store categorised sales into 72 categories, this study focused on fruit and vegetables, which fell over two till categories: ‘Fruit and Veg’ (fruit and vegetables purchased in packaging and sold at fixed prices) and ‘Fruit & Veg Weighed’ (fruit and vegetables purchased loose and sold by weight).

The data were then aggregated by week. Analysing and modelling using weekly data produced models that were both simpler to interpret and better according to the Ljung-Box test than models using daily data. In addition, long term effects, rather than day-to-day fluctuations, were of primary interest.

As would be expected, and from visually inspecting the data and discussions with store management, university term time and holiday periods were understood to have a major effect on sales across the store. For our analyses we included only weeks that fell wholly within term time. In addition it was noted that the final week of every 10 week term had lower sales of fruit and vegetables, which is likely due to students who plan to return home for the holiday periods reducing their purchases of perishable food items. For this reason we created a dummy variable indicating the last week of every term.

### Statistical analysis

The study was conducted as a retrospective interrupted time series analysis using dynamic regression. Interrupted time series analysis is a method for evaluating interventions that take place at a well-defined time point; it is particularly useful for evaluating interventions for which a randomised controlled trial is difficult or impossible, such as interventions which work at the level of a population, and for evaluating interventions retrospectively [33]. This study analyses the effect of an intervention on a population (the customers of a university grocery store) and was conceived and carried out after the intervention had taken place (i.e. was retrospective).

Initially, a table of summary statistics was produced. One-way Analysis of Variance (ANOVA) tests were undertaken to determine whether to reject the null hypothesis of no difference in mean total sales, mean fruit and vegetable sales and proportional fruit and vegetable sales between the intervention periods.

The data frame was converted into a time series object in R using the xts package [34]. Scatterplots of total daily sales over time, daily sales of fruit and vegetables and sales of fruit and vegetables were made using the R package ggplot2 [35]. At this point data were interpreted visually.

A dynamic regression model was used to model the total sales and the sales of fruit and vegetables. The intervention periods were entered into the model using binary (‘dummy’) variables. Specifically, the model used was a multiple regression model with an Auto Regressive Integrated Moving Average (ARIMA) model for the errors. The regression component captures trends and dependence on other variables (the ‘external regressors’). It was observed that sales data periods close together in time had sales more similar than would be expected at random (i.e. autocorrelation). To capture this autocorrelation, an ARIMA model was used for the errors. ARIMA models are commonly used for time series data (e.g. econometric data) which show autocorrelation. A subtlety here is the inclusion of time as a regressor. ARIMA errors can capture some types of trend over time (i.e. those due to drift). However, time was also included as a regressor as beyond an effect of drift over time it might be expected that there is some underlying effect of time (e.g. due to national trends in fruit and vegetable consumption). We also included the dummy variable indicating the last week of term as a regressor. To assess the appropriateness of the model used, another analysis was performed by producing a linear model with no ARIMA component. R^2^ calculations indicated that these models were not preferable to the dynamic regression models used.

It was then decided to model the proportion of total sales that were fruit and vegetables, also using dynamic regression. This was to deal with the observation that the overall total quantity of items bought fluctuated greatly within the period of study, and this may mask a differential effect on fruit and vegetables. In particular, in discussion with store management, there was concern that building works on the plaza area outside the store, which led to changes in bus routes and bus stop positions, affected total sales at several points in time during the study period. Using a linear model, rather than a generalised linear model, is often considered inappropriate when the outcome variable is a proportion, as proportions are limited to between 0 and 1. However, the relationship may be close to linear away from 0 and 1. In particular, a linear form was considered to give an appropriate approximation here as all values fell within a relatively narrow range of 0.035 to 0.08 (and all but one between 0.045 and 0.08) and we are not seeking to extrapolate beyond the data. Therefore, it was felt that the distorting effects of the boundaries at 0 and at 1 could be safely ignored.

The R package forecast was used [36]. In particular, the auto.arima function was used to select the best model, with the noted external regressors specified. When external regressors are included, auto.arima uses regression first to take into account the external regressors, then models the errors using an ARIMA model i.e. produces a dynamic regression model. The package automatically tests for seasonality, and would in this case return a seasonal model. This was considered sufficient, and although seasonality can be forced, as auto.arima tests sometimes fail to pick up on genuine seasonality, this was not considered necessary (once aggregated by week, the data did not show any great seasonality from visual inspection).

A Ljung-Box test was used to test the residuals from the dynamic regression model (this is a portmanteau test that has as null hypothesis that the autocorrelations of a time series are zero). The variance inflation factor was also calculated to check for problematic multicollinearity requiring caution in interpreting the model.

All analyses were carried out both for sales-by-quantity and for sales-by-money. Both metrics have disadvantages: Sales-by-quantity, is of interest from a public health perspective. However, there is some risk of masking or pronouncing an effect, if average pack size bought, for example, changes over the study period; Sales-by-money avoids this but may introduce problems if the price or relative price of fruit and vegetables compared with other items on sale changes over the study period. By examining the patterns in both models and checking for consistency, we can be more confident that there is a genuine underlying result.

## Results

Weeks that fell fully within term time between 09/01/2012 and 02/07/2017 were included in the final dataset. This included a total of 170 weeks of data: 90 weeks in the baseline period (09/01/2012–07/12/2014); 40 weeks during intervention A (05/01/2015–20/03/2016); and 40 weeks during intervention B (25/04/2016–02/07/2017).

**Fig. 1:**
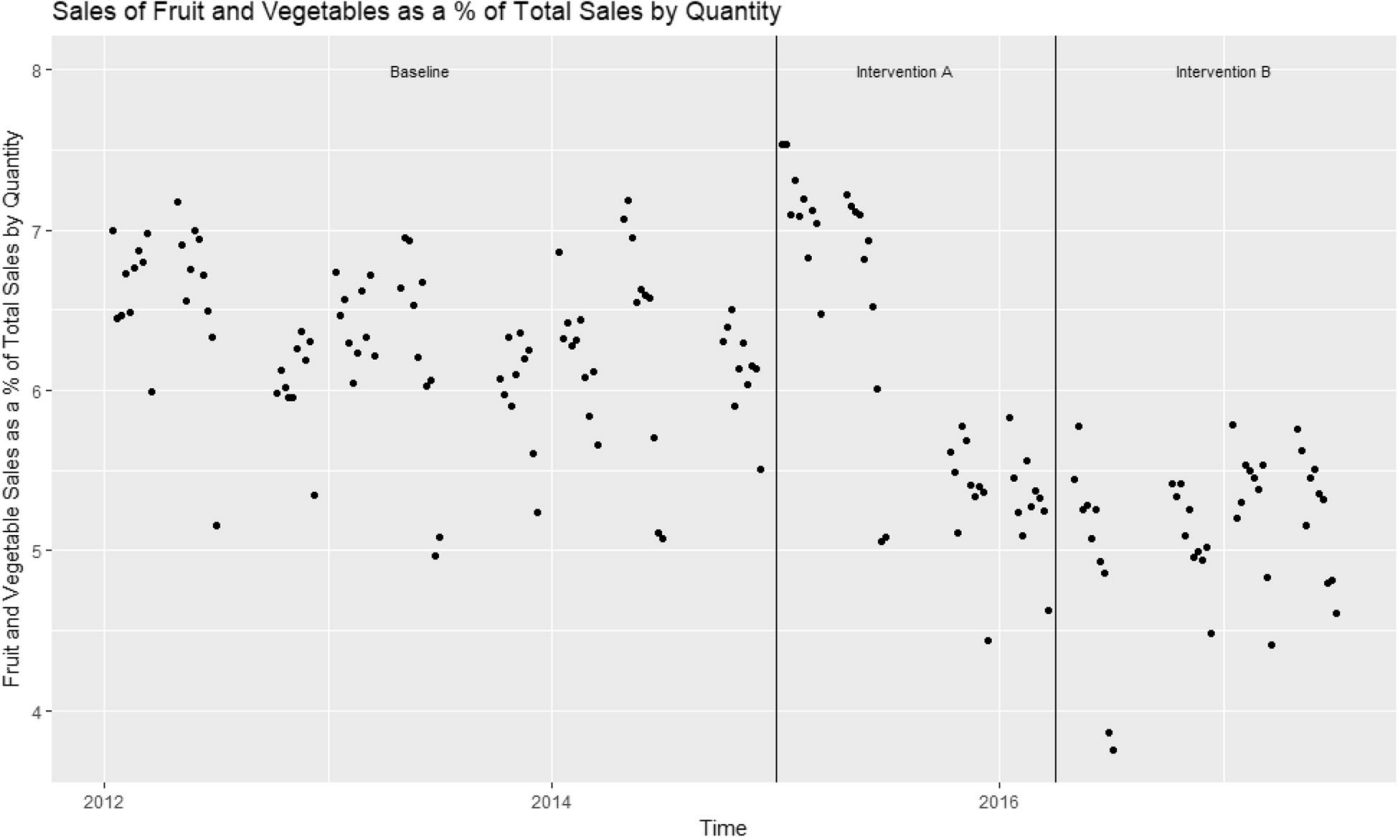
Sales of fruit and vegetables as a percentage of total sales by quantity

**Fig. 2:**
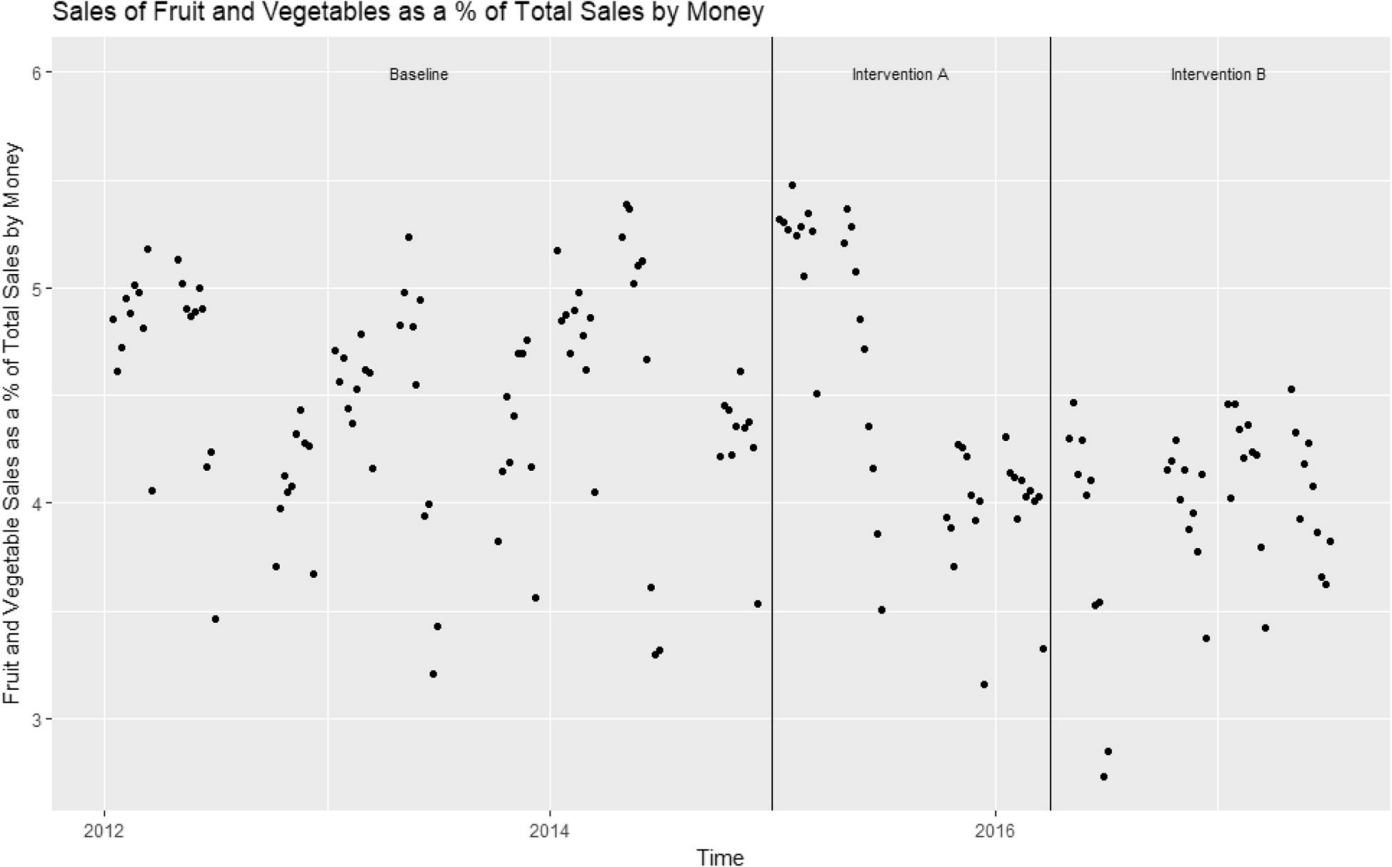
Sales of fruit and vegetables as a percentage of total sales by money

Figures 1 and 2 show scatterplots of percentage of sales that were fruit and vegetables, by quantity and by money respectively. In both figures there is an apparent downward trend in the percentage of sales which are fruit and vegetables across the time period, with a perceivable upswing in the period of Intervention A. It is also clear that in the last week of each 10 week term, there is a lower percentage of fruit and vegetable sales than in the previous 9 weeks.

There were just over 93,000 sales on average per week across the time period, of which 5564 (5.97%) were fruit and vegetable sales (Table 1). ANOVA tests demonstrated highly significant (*p* < 0.001) differences in the percentage of total sales that were fruit and vegetables between the time periods relating to baseline, intervention A and intervention B (Table 1). The percentage of total sales that were fruit and vegetables fell from 6.29% at baseline, to 6.07% during the intervention A time period and then to 5.23% during implementation of intervention B (Table 1).

**Table 1:** Characteristics of the data and univariate analyses of sales data per intervention period

	Mean Weekly Sales by Quantity	95% CI of Mean Weekly Sales by Quantity	Mean Weekly Sales by Money Taken (£)	95% CI of Mean Weekly Sales by Money Taken	Results of ANOVA: *p* value
Total Sales	93,074	91,205.38, 94,941.87	140,515	137,767.5, 143,261.4	
Total Sales, baseline	91,619	89,350.98, 93,887.44	140,605	137,145.4, 144,063.7	By Quantity *p* = 0.002 By Money *p* = 0.02
Total Sales, Intervention A	90,048	86,834.36, 93,261.56	134,812	130,113.7, 139,509.5	
Total Sales, Intervention B	99,372	94,456.39, 104,287.06	146,015	138,695.8, 153,333.5	
Fruit and Vegetables	5564	5412.71, 5714.431	6158	5983.119, 6333.611	
Fruit and Vegetables, baseline	5790	5597.108, 5983.692	6349	6113.253, 6584.247	By Quantity *p* = 0.003 By Money *p* = 0.07
Fruit and Vegetable, Intervention A	5464	5152.136, 5776.564	6005	5655.987, 6353.674	
Fruit and Vegetables, Intervention B	5152	4816.497, 5488.353	5884	5485.972, 6281.095	
% Fruit and Vegetables	5.97	5.853301, 6.086766	4.37	4.280872, 4.451047	
% Fruit and Vegetables, baseline	6.29	6.189831, 6.397652	4.50	4.388787, 4.601919	By Quantity *p* < 0.001 By Money *P* < 0.001
% Fruit and Vegetables, Intervention A	6.07	5.77514, 6.365298	4.45	4.238621, 4.655525	
% Fruit and Vegetables, Intervention B	5.23	4.996114, 5.286894	3.99	3.863621, 4.123804	

The dynamic regression models demonstrate a significant downward trend in the percentage of sales which were fruit and vegetables by quantity and by money across the whole time period. Both models additionally show a significant increase in the percentage of sales which were fruit and vegetables by quantity and by money during the intervention A time period, and an increase in the percentage of sales which were fruit and vegetables by quantity and by money during the intervention B time period that was not significant (Tables 2 and 3). In terms of the percentage of total sales which were fruit and vegetables by quantity, during Intervention A this increased by 0.97 percentage points and in terms of the percentage of total sales which were fruit and vegetables by money taken, during Intervention A this increased by 0.69 percentage points.

**Table 2:** Results of the dynamic regression model for percentage of total sales which were fruit and vegetables by quantity (weekly)

	Co-efficient	95% CI
Intervention A	0.97	0.34, 1.64
Intervention B	0.83	−0.11, 1.76
Time (per day)	−0.0012	−0.002, − 0.0004
Last week of term	− 0.84	−0.98, − 0.70
Constant	6.84	6.14, 7.54
AR1	1.44	1.12, 1.76
AR2	−0.47	−0.75,-0.18
MA1	−0.78	−1.03, − 0.52

**Table 3:** Results of the dynamic regression model for percentage of total sales which were fruit and vegetables by money taken (weekly)

	Co-efficient	95% CI
Intervention A	0.69	0.16, 1.22
Intervention B	0.71	−0.07, 1.49
Time (per day)	−0.0008	−0.001, − 0.0002
Last week of term	− 0.72	−0.84, − 0.61
Constant	4.92	4.51, 5.33
AR1	0.77	0.66, 0.87

For both models, the Ljung-Box test was significant, therefore the plots of autocorrelation function for the residuals left when fitting the models of percentage sales were inspected (Additional file 1: Figure S1 and Additional file 2: Figure S2). Inspection of the residuals did not give further cause for concern: there is no discernible patterning (e.g. from a seasonality effect not taken into account).

## Discussion

The effect of the shop re-arrangement to make fruit and vegetables more prominent (Intervention A) increased the percentage of total sales that were fruit and vegetables, when analysed by either items purchased or money spent. The effect of Intervention B, which was the same as Intervention A but for the replacement of a display of fruit and vegetables directly facing the entrance to the shop with a chiller cabinet, is less clear. It had a positive coefficient (compared to baseline) for both percentage sales by quantity and percentage sales by money, but this indicated increase was no longer significant at the 95% level. Therefore, it is likely, though not highly likely, that the choice architecture presented in Intervention B boosted percentage sales of fruit and vegetables compared with baseline.

The second, more concerning, major result of this study is the observed effect of time. The coefficient of time in the best fit model is negative in percentage fruit and vegetable sales by quantity, percentage fruit and vegetable sales by money and absolute fruit and vegetable sales by quantity, indicating a decrease in sales over time. In particular, the decline in percentage sales by money and sales by quantity was significant.

The lines (ie: types of products) stocked by the shop did not change greatly over the study period, meaning this likely represents a decline in the percentage of purchases being fruit and vegetables amongst the population making up the store customers.

Our first major result is supported by previous choice architecture literature suggesting that layout changes can positively influence buying behaviour such that more healthy food is bought. In a systematic review of interventions involving changing food placement on food choices, manipulating the order in which food products are encountered or the proximity of food options were found to influence purchasing behaviour [30]. In a systematic review of choice architecture interventions to improve healthy eating among healthcare personnel, similar ‘proximity’ interventions, such as arranging displays to make healthier products easier to access were found to be an effective way to ‘nudge’ consumers into making healthier purchases [37]. Other choice architecture interventions such as providing nutrition labelling and increasing the availability of healthy food, increased healthier food choices in tertiary education settings [29]. Universities should consider how to encourage campus retailers to use these techniques to promote healthy behaviour by their staff and students, perhaps through contract agreements.

In terms of the second major result, there is some indication it may be evidence of a more general trend. Health Survey for England data indicate that amongst 16 to 24 year olds, mean portions per day of fruit and vegetables have been on a monotonic downward trend since 2006 from 3.1 to 2.9 in 2015 (although taking into account standard errors, these values may not differ) [8]. There are other possible explanations: perhaps fruit and vegetables became more expensive in this shop compared to other local shops over the study period, for example, and the population chose to buy fruit and vegetables elsewhere. Due to the potentially serious consequences of declining fruit and vegetable consumption in this age group for public health, this observation merits further investigation.

The principal strength of this study is it examines a ‘natural experiment’: changes made to store layout as a normal part of store operations that affected the location of fruit and vegetables. Moreover, purchasing data were collected and stored as a normal part of store operations. This offered the opportunity for a long follow up and meant that historical data could be used and analysed, rather than having a researcher observe participants or any other design that might make it evident that purchases were being observed. Hence, no one purchasing food in the store was aware of the fact that their purchases would be analysed in order to assess fruit and vegetable purchasing behaviour.

Another advantage of this study is the volume of data collected. Data were collected from every purchase made in store from January 2012 to July 2017. This gives a long period in each intervention stage. This is advantageous as many studies report follow ups of a few months at most [29]. It may be the case that an effect is not sustained long term: a behaviour change may be triggered by the novel layout, but as consumers become aware of their new purchasing behaviours or used to the new environment it may wane. In examining the scatterplots we may see some waning of the effect. However, the fact that the dynamic regression models indicate a boost of both interventions, and statistically significantly so for intervention A, indicates some maintenance of the effect of the change. Nonetheless, the issue of whether the effect of a choice architecture ‘proximity’ change wanes over time merits further investigation and awareness.

This study has many limitations associated with its design. In particular, the fact the data are from a ‘natural experiment’ means it was not possible to adequately control for other factors that may have affected purchasing behaviour as would have been possible in a cluster randomised controlled trial. Some of these could be taken into account, or dismissed as only having minor effects on the results from discussions with store management. For example, the effect of university term time, other changes in store (lines stocked, price inflation). Others, however, such as the building work, may have had an effect on the results.

Building works taking place in 2014 and 2015 on the campus, involved many changes taking place over a period of months, with differing effects on footfall, including movement of a bus-stop previously located outside the store, which the store manager believed had resulted in reduced footfall at the shop. For this reason we chose to model percentage of total sales that were fruit and vegetables rather than absolute sales of fruit and vegetables. It may be argued that it is absolute, not percentage, sales of fruit and vegetables that are of interest from a public health perspective. However, as overall total sales fell around the time that Intervention A was implemented (likely due to the change in position of a bus-stop) any association between intervention period and absolute sales were likely to be attributable to confounding factors.

## Conclusions

The main conclusion of this study is that a change to store layout can boost purchasing of fruit and vegetables in a UK tertiary education setting, and this effect appears to be maintained over time. This is therefore a viable method of improving the nutritional quality of diets in this population and should be considered by universities when designing retail facilities on campus or contracting private retailers.

A second conclusion is that a trend of declining fruit and vegetable purchasing over the five and a half year study period was seen. This merits further investigation to examine whether this effect is repeated more widely. If so, it represents a threat to public health, and the factors driving it ought to be examined and understood in order that it might be tackled and reversed.

## Additional files

Additional file 1: **Figure S1.** Plot of autocorrelation function for the residuals left when fitting the models of percentage sales by quantity. (PPTX 36 kb)

Additional file 2: **Figure S2.** Plot of autocorrelation function for the residuals left when fitting the models of percentage sales by money. (PPTX 41 kb)

Additional file 3: **Figure S3.** Floor plan of the Rootes grocery store before the intervention and after the intervention. A highlights the location of the fruit and vegetables during the baseline period on the “before” plan. During Intervention A fruit and vegetables were situated in areas B and C, and during Intervention B fruit and vegetables were situated in area B only. (PDF 17 kb)

## Abbreviations

ARIMA: Auto Regressive Integrated Moving Average
WHO: World Health Organisation
