# Supplementary figures and images for: Choice architecture modifies fruit and vegetable purchasing in a university campus grocery store: time series modelling of a natural experiment
Source: BMC Public Health. 2018 Oct 1;18:1149. doi: 10.1186/s12889-018-6063-8 (PMC6167822; doi:10.1186/s12889-018-6063-8)

## Slide 1
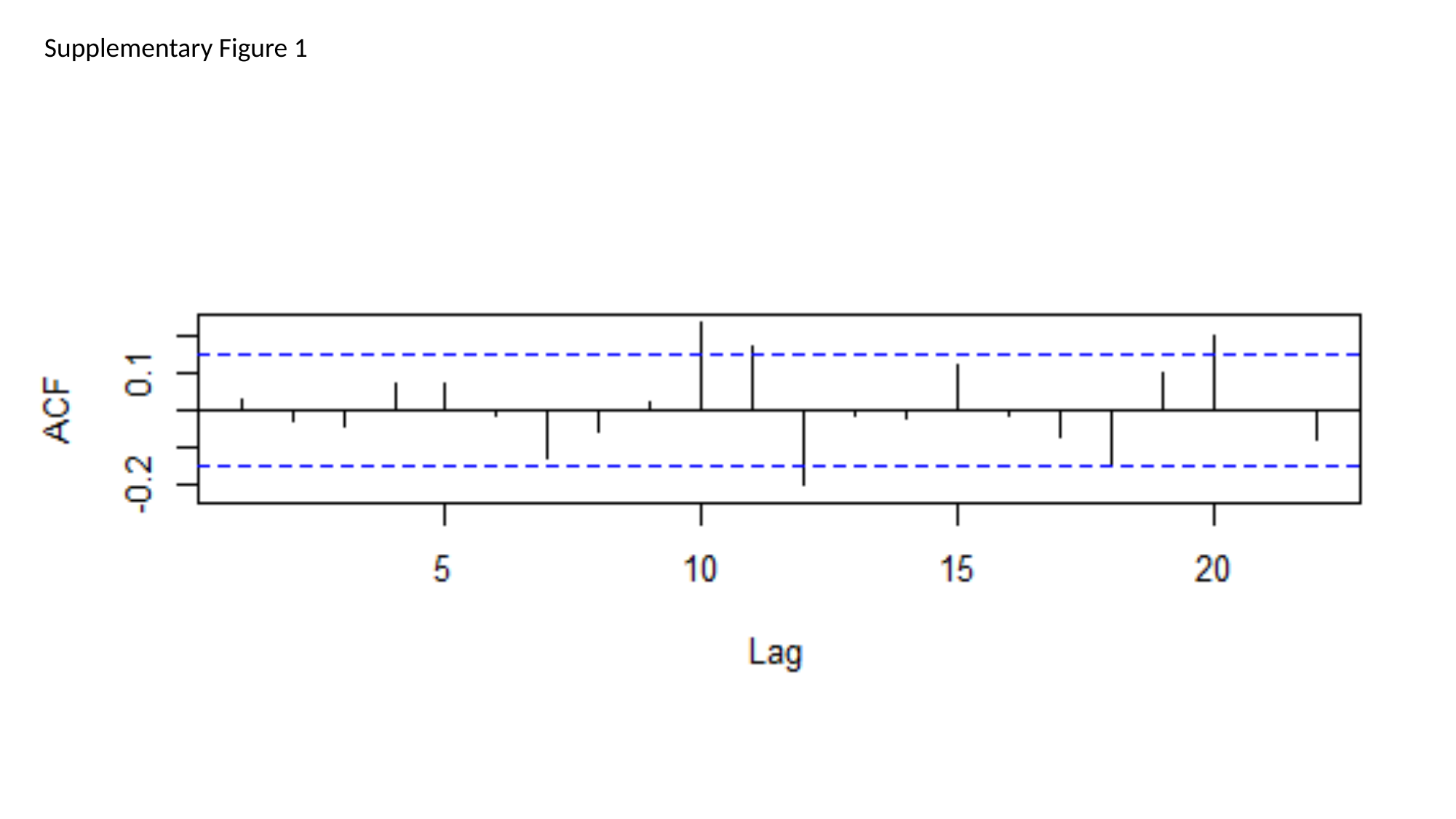

Supplementary Figure 1

Supplement: Supplementary file 1 — Figure S1. Plot of autocorrelation function for the residuals left when fitting the models of percentage sales by quantity. (PPTX 36 kb) [file 12889_2018_6063_MOESM1_ESM.pptx]

## Slide 1
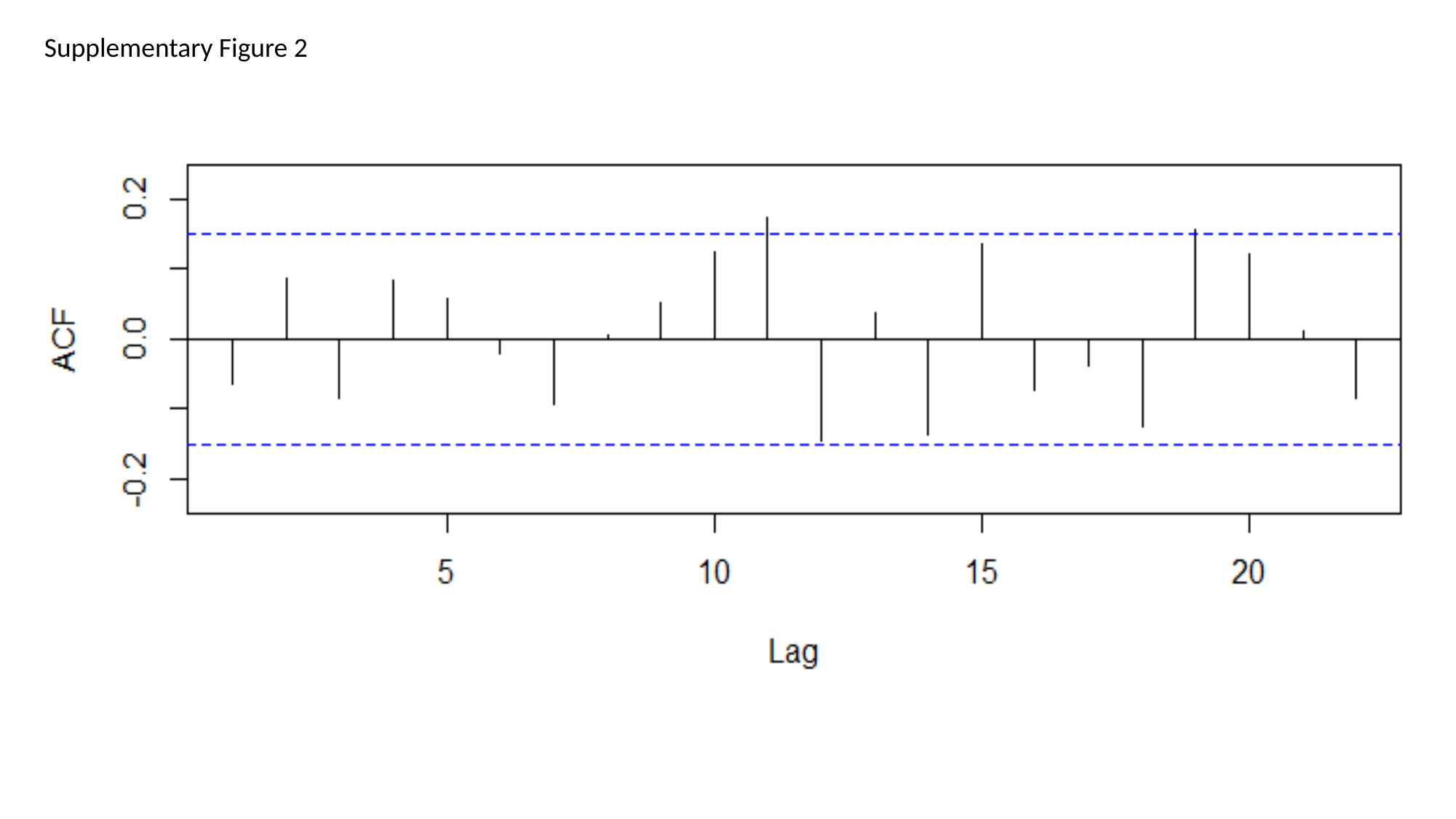

Supplementary Figure 2

Supplement: Supplementary file 2 — Figure S2. Plot of autocorrelation function for the residuals left when fitting the models of percentage sales by money. (PPTX 41 kb) [file 12889_2018_6063_MOESM2_ESM.pptx]

# Before

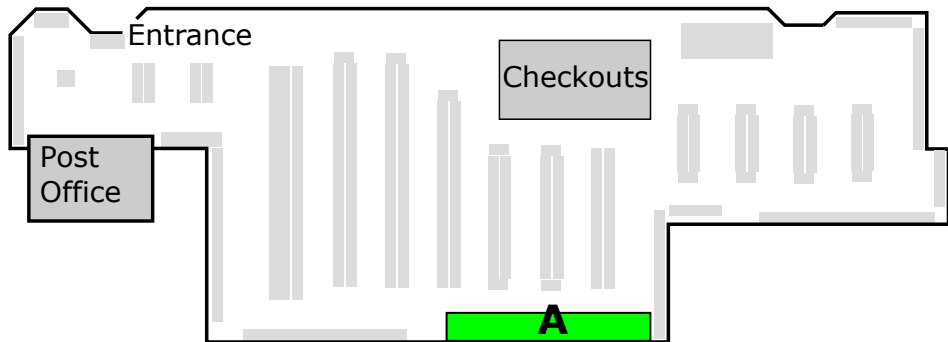

# After

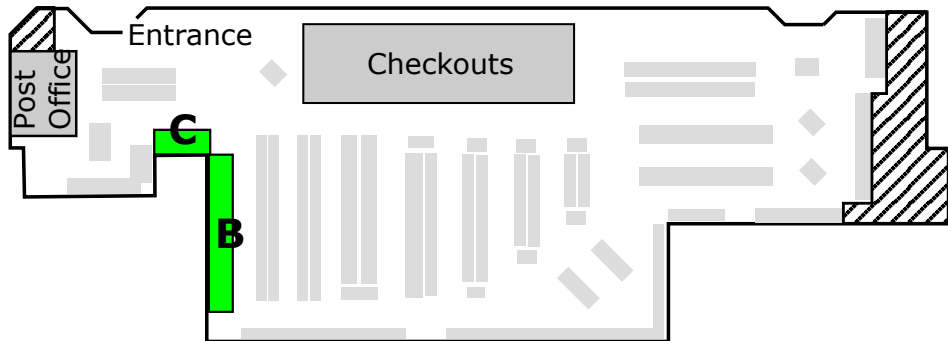

Supplement: Supplementary file 3 — Figure S3. Floor plan of the Rootes grocery store before the intervention and after the intervention. A highlights the location of the fruit and vegetables during the baseline period on the “before” plan. During Intervention A fruit and vegetables were situated in areas B and C, and during Intervention B fruit and vegetables were situated in area B only. (PDF 17 kb) [file 12889_2018_6063_MOESM3_ESM.pdf]
